# Supplementary material for: A Transparency Sheet-Based Colorimetric Device for Simple Determination of Calcium Ions Using Induced Aggregation of Modified Gold Nanoparticles
Source: Int J Mol Sci. 2019 Jun 17;20(12):2954. doi: 10.3390/ijms20122954 (PMC6627648; doi:10.3390/ijms20122954)
Supplement: Supplementary file 1 [file ijms-20-02954-s001.pdf]

Supplementary Information

A Transparency Sheet-Based Colorimetric Device for Simple Determination of Calcium Ions using Induced Aggregation of Modified Gold Nanoparticles

Paweenar Duenchay [1], Orawon Chailapakul [2] and Weena Siangproh [1,*]

[1] Department of Chemistry, Faculty of Science, Srinakharinwirot University, Sukhumvit 23, Wattana, Bangkok 10110, Thailand

[2] Electrochemistry and Optical Spectroscopy Centre of Excellence (EOSCE), Department of Chemistry, Faculty of Science, Chulalongkorn University, Patumwan, Bangkok 10330, Thailand

* Correspondence: weena@g.swu.ac.th; Tel.: +662 649 5000 ext.18208

Table S1. Zeta potential measurement of AHMP, AHMP-AuNPs, Buffer-AHMP-AuNPs and Ca^{2+}-Buffer-AHMP-AuNPs.

Samples Name	Z-Average (nm)	Zeta Potential (mV)	Intensity (a.u.)
AuNPs	26.9	−43.1	32
AHMP-AuNPs	29.1	−49.9	38
Buffer-AHMP-AuNPs	27.6	−58.2	35
Ca^{2+}-Buffer-AHMP-AuNPs	65.4	−50.3	26

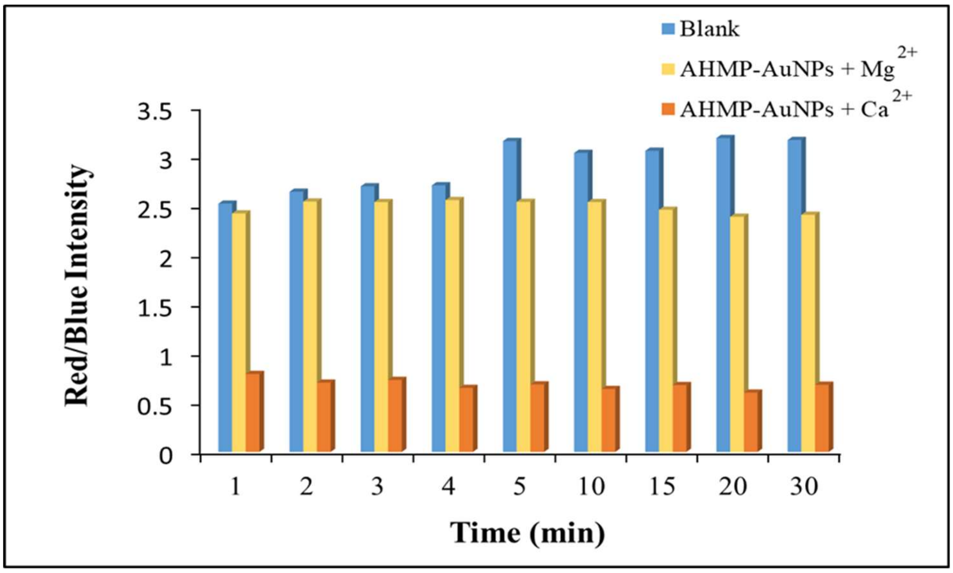

Figure S1. The Effect of the incubation time for the aggregation of AHMP-AuNPs without and with Ca^{2+} 100 ppm compare with Mg^{2+} 100 ppm, phosphate buffer pH 6, AHMP-AuNP: sample volume ratio at 1:1.

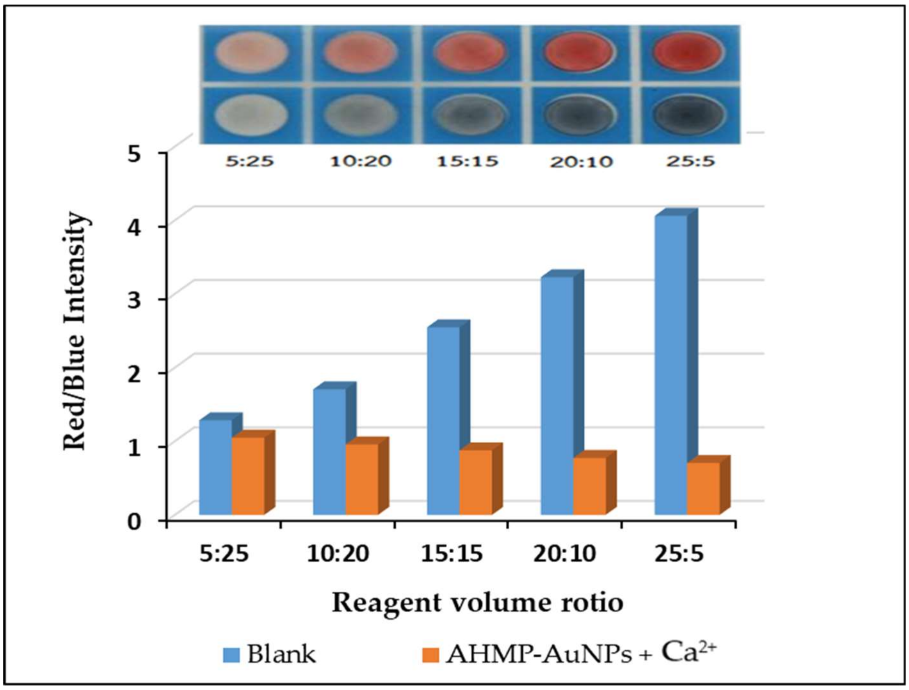

Figure S2. The Effect of the reagent volume ratio for the aggregation of AHMP-AuNPs and Ca^{2+} at 100 ppm, incubation time 1 min, Other conditions used as same as optimal values.

Table S2. Tolerance of AHMP-AuNPs with interference as Ca^{2+} 100 ppm.

**Ca^{2+}:Interferences**	**Ratio of color change) Tolerance)**
Ca^{2+}:Ascorbic acid	1:20
Ca^{2+}: Glucose	1:200
Ca^{2+}: Uric acid	1:40
Ca^{2+}: Albumin	1:200
Ca^{2+}:Cd^{2+}	1:15
Ca^{2+}:Cu^{2+}	1:10
Ca^{2+}:Hg^{2+}	1:10
Ca^{2+}:Zn^{2+}	1:10
Ca^{2+}:Mg^{2+}	1:10
Ca^{2+}:K^{+}	1:30
Ca^{2+}:Na^{+}	1:40
Ca^{2+}:Cl^{-}	1:40
Ca^{2+}:SO_4^{2-}	1:10
Ca^{2+}:CO_3^{-}	1:20
Ca^{2+}:PO_4^{2-}	1:10

Table S3. Comparison of analytical performances of different methods for the determination of Ca^{2+}in biological fluid.

Method	Sample	LOD (ppm)	LOQ (ppm)	Linear range (ppm)	Ref.
Atomic absorption spectrometry (AAS)	Blood	0.02	0.07	0.02–2.60	8
Sequential injection analysis/ Spectrophotometer	Pharmaceutical, Water, Urine	0.05	-	0–20	11
Atomic emission spectrometry (AES)	Gluconates oral solution, Blood	0.01–0.10	-	1–10	13
Fluorescence	Human serum	0.64	1.72	1.72-8.02	15
Fluorescence	Human serum	3.09×10^{-6}	-	$4 \times 10^{-5} – 4 \times 10^{-4}$	16
Fluorescence	Blood	10.00	-	0–80,000	17
Ion selective electrode/ electrochemistry	Blood serum	0.48	-	20.04–32.06	24
Transparency sheet-based colorimetric device	Urine	3.05	10.17	10–100	This work

Table S4. Determination of Ca^{2+} in artificial urine samples using the proposed method compared with an AAS method (n = 5).

Samples	Added Ca^{2+} (ppm)	AAS method		The proposed method	
		Found	%Recovery	Found	%Recovery
**Sample 1**	0	0.00 ± 0.00	-	0.00 ± 0.00	-
**Sample 2**	80	78.96 ± 1.80	98.7	83.40 ± 3.67	104.3
**Sample 3**	160	164.07 ± 0.00	102.5	166.31 ± 4.74	103.9
**Sample 4**	320	315.37 ± 3.37	98.6	331.81 ± 2.61	103.7
